# The Long Non-Coding RNA HOXC-AS3 Promotes Glioma Progression by Sponging miR-216 to Regulate F11R Expression

**DOI:** 10.3389/fonc.2022.845009

**Published:** 2022-03-23

**Authors:** Yongshuai Li, Lu Peng, Xianwen Cao, Kun Yang, Zhen Wang, Yong Xiao, Hong Xiao, Chunfa Qian, Hongyi Liu

**Affiliations:** ^1^ Department of Neurosurgery, Affiliated Nanjing Brain Hospital, Nanjing Medical University, Nanjing, China; ^2^ Department of Clinical Laboratory, Affiliated Nanjing Brain Hospital, Nanjing Medical University, Nanjing, China; ^3^ Department of Neuro-Psychiatric Institute, Affiliated Nanjing Brain Hospital, Nanjing Medical University, Nanjing, China

**Keywords:** HOXC-AS3, miR-216, F11R, ceRNA, glioma

## Abstract

HOXC cluster antisense RNA 3 (HOXC-AS3) is a long noncoding RNA (lncRNA) that plays a crucial role in various tumors; nevertheless, its role in glioma and its mechanism have not been completely elucidated. In this research, we discovered that HOXC-AS3 was over-expression in glioma cells and tissues and was associated with prognosis. Next, we determined that HOXC-AS3 targeted miR-216 as a sponge and that the F11 receptor (F11R) was the target of miR-216 by online databases analysis, qRT–PCR, and luciferase reporter assay. In addition, the rescue experiments confirmed that HOXC-AS3 regulated the expression of F11R by competitively binding miR-216 and functioning as a competing endogenous RNA (ceRNA). The intracranial glioblastoma mouse model suggested that HOXC-AS3 could promote glioma malignant progression *in vivo*. In summary, our study shows that the HOXC-AS3/miR-216/F11R axis plays an important role in the malignant progression of glioma, and may provide new ideas for the treatment of glioma.

## Introduction

Glioma, the most common primary malignant tumor, accounts for 51.4% of all primary cerebral tumors in the central nervous system (CNS) (1). Glioblastoma (GBM) is the most fatal glioma and the most common glioma in adults, with a median survival of only 14 months (2). To date, glioma is characterized by tumoral genetic heterogeneity, rapid proliferation, extensive migration, and invasion. It is difficult to achieve satisfactory outcomes with therapeutic schedules involving maximal safe surgical resection, radiotherapy, and chemotherapy (3). Thus, it is vital to identify new molecules with regulatory functions and related signaling pathways to elucidate the mechanism of glioma.

Noncoding RNAs (ncRNAs), particularly long noncoding RNAs (lncRNAs), play an important role in various types of cellular and biological functions (4). LncRNAs are defined as RNAs of more than 200 nucleotides that do not encode proteins. LncRNAs can be involved in multiple biological behaviors by regulating gene expression at the epigenetic, transcriptional, and posttranscriptional levels. An increasing number of studies have revealed that lncRNAs are connected to multiple cellular processes, including cell differentiation, proliferation, apoptosis, migration, invasion, and immunotherapy resistance in cancer (5–9).

The lncRNA HOXC cluster antisense RNA 3 (HOXC-AS3), which is located at 12q13.13, has been observed to act as an oncogene in a variety of tumors. For instance, Yang B et al. reported that HOXC-AS3 promotes the proliferation of ovarian cancer cells by suppressing mature miR-96 (10). Yang Z et al. found that the lncRNA HOXC-AS3 accelerates pulmonary invasive mucinous adenocarcinoma progression by regulating FUS/FoxM1 and is probably considered a therapeutic marker (11). Another study showed that HOXC-AS3 modulates the tumorigenesis of gastric cancer by binding YBX1 (12). In glioma, studies have shown that HOXB13 directly binds the promoter of HOXC-AS3. In addition, HOXB13 promotes the proliferation, migration, and invasion of GBM cells through HOXC-AS3 (13). However, the specific mechanism by which HOXC-AS3 regulates GBM cell function is still unclear, and its downstream signaling pathway remains unascertained. Accordingly, it is significant to probe the potential mechanism of HOXC-AS3 in glioma.

At present, our research demonstrates that the expression level of HOXC-AS3 in glioma tissues and cell lines is elevated than that in normal tissues and cells. Furthermore, we disclosed that HOXC-AS3 modulates F11 receptor (F11R) expression by sponging miR-216 and facilitating glioma cell proliferation, migration, invasion, and tumor growth *in vivo*. Collectively, these results reveal that the HOXC-AS3/miR-216/F11R signaling pathway is involved in the biological behavior of glioma and may serve as a novel potential target for the treatment of glioma.

## Materials and Methods

### Clinical Tissue Specimens

Twenty-three glioma specimens and fifteen normal brain tissues (NBTs) were obtained from the Department of Neurosurgery, the Affiliated Brain Hospital of Nanjing Medical University. The tissues were snap-frozen in liquid nitrogen and placed at -80°C for preservation after surgical resection. Ethical approval was granted by the ethics committee of the Affiliated Brain Hospital of Nanjing Medical University, and all patients signed written informed consent.

### Cell Culture

The glioma cell lines (LN229, T98G, A172, U87 and U251) were purchased from the Chinese Academy of Sciences Cell Bank (Shanghai, China), while the normal human astrocytes (NHAs) were acquired from ScienCell Research Laboratories (Carlsbad, CA, USA). The glioma cells were cultured in Dulbecco’s modified Eagle’s medium (DMEM, Gibco, USA) supplemented with 10% fetal bovine serum (ScienCell, USA), and the NHAs were cultured in astrocyte medium (Life Technologies MA, USA). All cells were cultured in an incubator containing 5% CO_2_ at 37°C.

### Cell Transfection

Short hairpin RNAs (shRNAs) targeting HOXC-AS3 (sh-HOXC-AS3-1 and sh-HOXC-AS3-2) and F11R (sh-F11R), overexpression plasmids targeting HOXC-AS3 (HOXC-AS3) and F11R (F11R), and their corresponding negative control RNAs (sh-NC) were designed by GenePharma (Shanghai, China). The miR-216 mimics, inhibitors, and relative controls were obtained from Sangon Biotech (Shanghai, China). The transfection assays were conducted using Lipofectamine 3000 (Invitrogen, CA, USA). All interfering nucleotides are shown in **Table S1**.

### RNA Isolation and Quantitative Real-Time PCR (qRT–PCR)

The total RNA was extracted from the specimens and cells using TRIzol (Vazyme, Nanjing, China) according to the manufacturer’s protocol. qRT–PCR was carried out to test the expression levels of HOXC-AS3, miR-216, and F11R. The data was normalized to U6 and GAPDH, and the expression level was analyzed using the 2^–ΔΔCt^ method. Reverse transcription (RT) was conducted according to the method specified in the instructions using a C1000 Touch Thermal Cycler (Bio–Rad, California, USA). The amplification reaction under predetermined conditions was performed using Applied Biosystems QuantStudio 5 (Thermo Fisher Scientific, MA, USA). Each experiment was conducted three times. All primers are shown in **Table S2**.

### Luciferase Reporter Assay

The fragment of HOXC-AS3 that contained the miR-216 binding site was inserted into GP-miRGLO vectors. The 3′-UTR fragments of F11R containing the binding sites for miR-216 were inserted into GV272 vectors. MiR-216 mimics and the relative control were transfected into the glioma cell lines with the reporter plasmids and mutated plasmids, respectively. After 48 hours, luciferase activity was observed using a Dual Luciferase Reporter Assay System (Promega, Madison, WI, USA). Sequences are shown in **Table S3**.

### Western Blot Assay

The total protein was isolated from the cells with RIPA buffer (YIFEIXUE BIO TECH, Nanjing, China). A BCA Protein Assay Kit (YIFEIXUE BIO TECH, Nanjing, China) was used to detect the protein concentration. SDS‐PAGE (12%) was used to resolve the protein, and after being transferred to PVDF membranes (Millipore, USA), it was blocked with skim milk (5%) for 2 h, washed three times with TBS-T, and then incubated at 4°C with primary antibodies against F11R (1:1000, Proteintech, IL, USA) overnight and HRP-conjugated secondary antibody (1:5000, Proteintech, IL, USA) for 2 hours. β-actin (1:1000, Proteintech, IL, USA) was used as a control.

### Fluorescence *In Situ* Hybridization (FISH)

The HOXC-AS3 probe sequences (**Table S4**) were synthesized by GenePharma (Shanghai, China), and the fluorescent signals of the probe were tested using a FISH Kit (GenePharma, Shanghai, China). Briefly, U87 and U251 cells were fixed in 4% paraformaldehyde for 15 min. Then were incubated at 37°C using 0.1% Triton X-100 (15 min) and 2× SSC (30 min). After that, the cells were hybridized in denatured probes (concentration of 1 μM) at 37°C overnight, washed for 5 min with 0.1% Tween 20, 5 min with 2× SSC, and 5 min with 1× SSC at 42°C. Ultimately, DAPI (Beyotime, China) was used to stain the cell nuclei. Images were captured under a Carl Zeiss microscope (Carl Zeiss, Germany).

### Cell Counting Kit-8 (CCK-8) Assay

U87 and U251 were seeded into 96-well plates at 10,000 cells/well overnight, cell proliferation was measured using a CCK-8 (KeyGEN Bio TECH, Nanjing, China) assay. Ten microliters of CCK-8 solution were added to each well, incubated at 37°C, 4 hours. The absorbance of each well was read at 450nm with a microplate reader (TECAN, Switzerland). Similarly, cell proliferation was detected after 24 h, 48 h, and 72 h, and then the cell growth curve was plotted.

### 5-Ethynyl-20-Deoxyuridine (EdU) Assay

Cells (75,000) were plated in 24-well plates with round coverslips overnight. 200 µl of EdU (10 µM, KeyGEN Bio TECH, Nanjing, China) were added to each well, and the cells were incubated at 37°C, 2 h. The cells were fixed by adding 4% neutral paraformaldehyde to each well and removed after 30 minutes at room temperature. Then, we added 0.5% TritonX-100 (KenGEN, Nanjing, China) in PBS (200 µl) to each well for 20 min and stained the samples with 200 µl Click-iT reaction mixture (KeyGEN Bio TECH, Nanjing, China) for 30 min under lightproof conditions. Next, DAPI (Beyotime, China) was used to stain the glioma cell nuclei. Round coverslips were observed using Carl Zeiss microscope (Carl Zeiss, Germany) to analyze the proportion of EdU-positive cells.

### Migration and Invasion Assays

To measure the ability of the cells to migrate and invade, Matrigel-covered (50 µl, 1:8 dilution, BD, USA) or uncovered Transwell insert chambers (Millipore, MA, USA) were utilized following the instructions. Then cells were collected and resuspended in serum-free medium and plated at 50,000 cells per upper chamber, while 500 µl medium with 10% FBS were added to the lower chambers. Forty-eight hours later, the residual cells in the upper chamber were removed. Simultaneously, penetrated cells were treated with paraformaldehyde and crystal violet (Beyotime, China). Three fields were randomly selected using the microscope (Nikon, Japan) to calculate the number of migrating and invading cells.

### Immunohistochemistry

Paraffin-embedded human and nude mouse glioma samples sections were incubated with primary antibodies against F11R (1:200, Proteintech, IL, USA) or Ki-67 (1:500 dilution, Servicebio, Wuhan, China) overnight at 4°C, and then incubated with the secondary antibody (1:200 dilution, Servicebio, Wuhan, China) at room temperature for 1 h. Next, the sections were stained with diaminobenzidine until brown precipitation appeared and counterstained with hematoxylin for 5 minutes. Positive areas of the captured image were analyzed by ImageJ.

### Terminal Deoxynucleotidyl Transfer-Mediated dUTP Nick End Labeling Staining (TUNEL)

After deparaffinization and rehydration, proteinase K was dropped onto the tissue sections for antigen retrieval. Following the instructions to block endogenous peroxidase, equilibrium was reached at room temperature, and the TUNEL reaction mixture was added (Servicebio, Wuhan, China). DAB development was then performed, and the nuclei were counterstained, followed by dehydration and mounting.

### Intracranial GBM Mouse Model

Male BALB/c nude mice were obtained from the Vital River (Beijing, China), 1 × 10^6^ U87 cells stably expressing EGFP-luciferase and transfected with sh-HOXC-AS3 or negative control were injected into the frontal lobes of nude mice for GBM orthotopic xenograft tumorigenesis (6 per group). The tumor volumes were measured 7, 14, and 21 days after the implantation using a bioluminescence imaging system (Caliper IVIS Spectrum, PerkinElmer, USA). D-Luciferin potassium salt (Beyotime, China) was injected intraperitoneally before the measurement. After the mice died, their brain tissue was removed and fixed with paraformaldehyde for further experiments.

### Statistical Analysis

The data was analyzed using SPSS 20.0 software (IBM, New York, USA) and is expressed as the mean ± standard error. The statistical significance was evaluated by using t-tests between two groups, one/two-way ANOVA and *post hoc* test (Bonferroni) among multiple groups. Log-rank test was used for survival analysis, and the relation between HOXC-AS3 and miR-216 as well as miR-216 and F11R was assessed by Pearson’s correlation analysis. P < 0.05 was considered statistical significance. All experiments were performed at least three times independently.

## Results

### HOXC-AS3 Expression Is Upregulated in Both Glioma Tissues and Cell Lines

**Figure 1:**
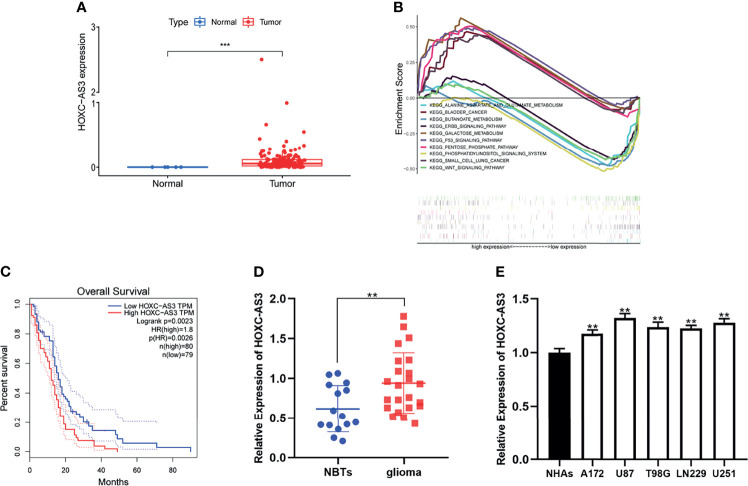
HOXC-AS3 was upregulated in glioma and associated with poor prognosis. **(A)** The expression level of HOXC-AS3 in TCGA. **(B)** HOXC-AS3 associated pathways were investigated using gene set enrichment analysis (GSEA) by TCGA genes data. **(C)** The Survival Plots of HOXC-AS3 in GEPIA. **(D)** Expression of HOXC-AS3 in 15 normal brain tissues and 23 glioma tissues. **(E)** Expression of HOXC-AS3 in normal human astrocytes and glioma cell lines. Mann-Whitney test for **(A)**; Log-rank test for **(C)**; t-test for **(D)**; one-way ANOVA and *post hoc* test for **(E)**. ***P < 0.001, **P < 0.01.

First, we evaluated the expression level of HOXC-AS3 using RNA sequencing data of 169 glioma tissues and 5 normal tissues in The Cancer Genome Atlas (TCGA) (https://portal.gdc.cancer.gov/) and found that HOXC-AS3 expression was markedly increased in GBM tissues compared to that in normal tissues (P < 0.05) (**Figure 1A**). Then, by conducting a GSEA (gene set enrichment analysis) (http://www.broadinstitute.org/gsea/index.jsp), we found that HOXC-AS3 may be related to some pathways involved in tumor metabolism (**Figure 1B**). Furthermore, we searched the TCGA dataset from GEPIA (http://gepia.cancer-pku.cn/index.html) (14), and the survival analysis indicated that the HOXC-AS3 expression level was inversely correlated with the overall survival of GBM patients (**Figure 1C**).

We also collected clinical samples during surgery, tested the expression of HOXC-AS3 in glioma and normal brain tissues (15) by qRT–PCR and found that the expression level of HOXC-AS3 was similar to that predicted by online databases (**Figure 1D**). Then, we examined the expression of HOXC-AS3 in normal human astrocytes (NHAs) and glioma cell lines (LN229, T98G, A172, U87, and U251). The results demonstrated that the expression level of HOXC-AS3 in the glioma cell lines was elevated than that in the NHAs (**Figure 1E**).

### HOXC-AS3 Promotes Glioma Cell Proliferation, Migration, and Invasion *In Vitro*

To analyze the effect of HOXC-AS3 in glioma, U87 and U251 cells were transfected with a shRNA targeting HOXC-AS3 (sh-HOXC-AS3-1 and sh-HOXC-AS3-2). The transfection efficiency was determined by qRT–PCR, and sh-HOXC-AS3-1 had better knockdown efficiency and was used for subsequent research (**Figures S1A, B**). Cell proliferation was identified by EdU and CCK-8 assays, and the migration and invasion abilities were assessed by Transwell assays. The results indicate that the functions of sh-HOXC-AS3 include inhibiting glioma cell proliferation, migration, and invasion *in vitro* (**Figures S1C–L**). In contrast, the upregulation of HOXC-AS3 showed the opposite results (**Figures S2A–L**). These results show that HOXC-AS3 acts as an oncogene that affects the biological processes of glioma cells *in vitro*.

### HOXC-AS3 Directly Targeted miR-216 as a Sponge

**Figure 2:**
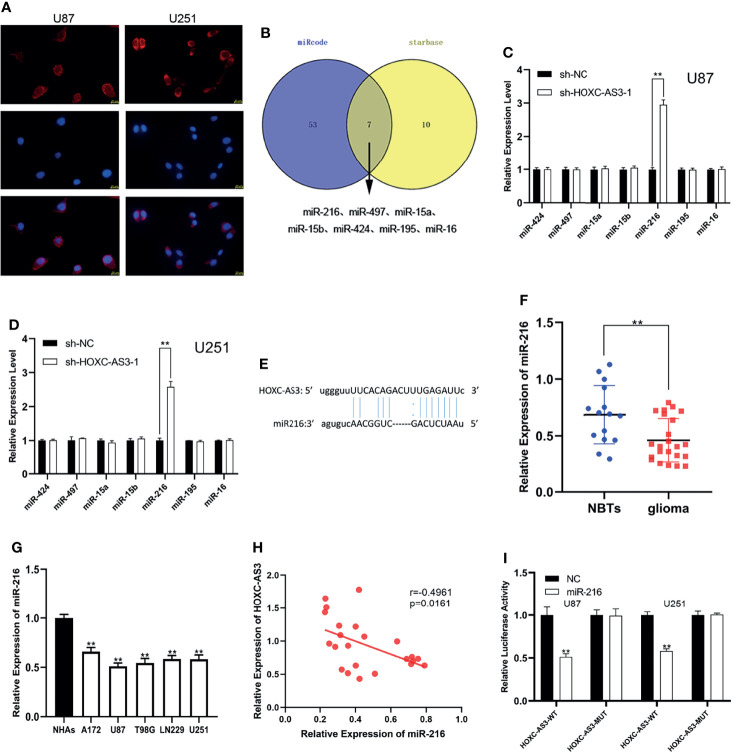
HOXC-AS3 acts as a sponge for miR-216. **(A)** The distribution of HOXC-AS3 in U87 and U251 cells was evaluated by FISH assays. **(B)** Targets of HOXC-AS3 were predicted by online databases. **(C, D)** The expression of seven miRNAs was detected in glioma cells transfected with sh-HOXC-AS3-1 or shNC. **(E)** Binding sites of miR-216 on HOXC-AS3. **(F)** Expression of miR-216 in normal brain tissues and glioma tissues. **(G)** Expression of miR-216 in glioma cell lines. **(H)** Pearson correlation analysis showed that HOXC-AS3 was inversely correlated with miR-216 in glioma tissues. **(I)** The association between miR-216 and HOXC-AS3 was verified by luciferase reporter assay. Pearson’s correlation analysis for **(H)**; t-test for **(C, D, I)**; Mann-Whitney test for **(F)**; one-way ANOVA and *post hoc* test for **(G)**. **P < 0.01.

Recently, numerous studies have confirmed that lncRNAs can serve as molecular sponges of miRNAs, thereby playing a role in regulating cell functions (16–18). We speculate that HOXC-AS3 may function through a competing endogenous RNA (ceRNA) pathway, i.e., HOXC-AS3 binds miRNAs to modulate the cell progression of glioma. Through a FISH analysis, we found that HOXC-AS3 was predominantly expressed in the cytoplasm of glioma cells (**Figure 2A**). This result further confirms our hypothesis that HOXC-AS3 functions through a ceRNA mechanism. Accordingly, we used the online databases starBase (http://starbase.sysu.edu.cn/) and miRcode (http://www.mircode.org/) to identify miRNAs that might interact with HOXC-AS3. We identified seven possible biological target miRNAs of HOXC-AS3 (**Figure 2B**). The expression of the target miRNAs in the U87 and U251 cells transfected with sh-HOXC-AS3 and NC was analyzed by qRT–PCR. The results showed that miR-216 expression was the most notably upregulated (**Figures 2C, D**). Potential miR-216 binding sites in HOXC-AS3 transcripts were identified by using starBase (**Figure 2E**). Furthermore, miR-216 was downregulated in glioma as revealed by qRT–PCR (**Figure 2F**). We tested the expression of miR-216 in five glioma cell lines and NHAs, and the results showed that miR-216 was underexpressed in glioma cells compared to NHAs (**Figure 2G**). Moreover, Pearson correlation analysis indicated that HOXC-AS3 was inversely correlated with miR-216 (**Figure 2H**). Finally, luciferase reporter plasmids were constructed including HOXC-AS3-WT and HOXC-AS3-MUT. The results indicated that miR-216 overexpression markedly reduced the luciferase activity of HOXC-AS3-WT without affecting the luciferase activity of HOXC-AS3-MUT (**Figure 2I**). These findings suggest that HOXC-AS3 directly targets miR-216 as a sponge.

### MiR-216 Inhibits Glioma Progression by Regulating F11R Expression

**Figure 3:**
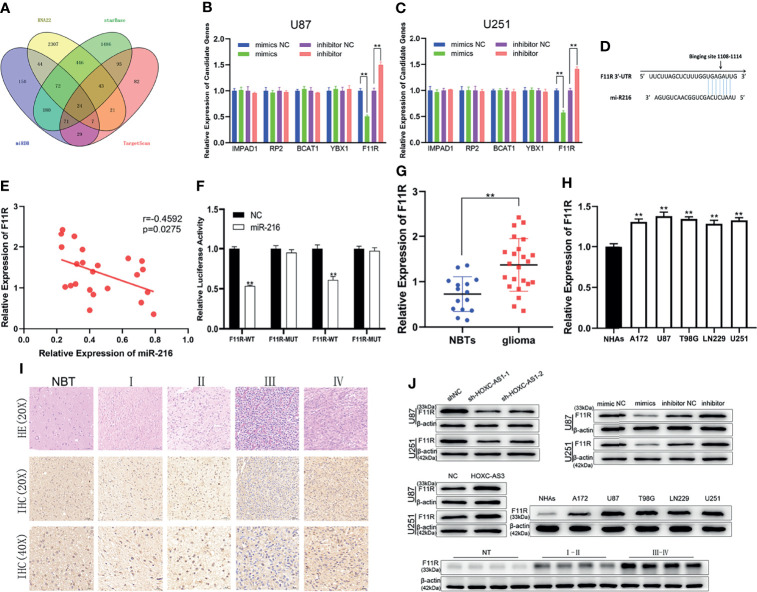
| MiR-216 directly binds F11R to regulate the level of F11R. **(A)** 24 potential downstream genes of miR-216 were predicted using online databases. **(B, C)** The effect of miR-216 on the expression of candidate mRNAs in U87 and U251 cells was detected by qRT-PCR. **(D)** Binding sites of miR-216 on F11R. **(E)** Pearson correlation analysis showed that F11R was inversely correlated with miR-216 in glioma tissues. **(F)** The association between miR-216 and F11R was verified by luciferase reporter assay. **(G)** Expression of F11R in normal brain tissues and glioma tissues. **(H)** Expression of F11R in normal human astrocytes and glioma cell lines. **(I)** Expression of F11R in different grades of glioma identified using immunohistochemistry. **(J)** The expression levels of F11R in different tissues and cell lines were tested by western blot analysis. At the same time, it was verified that F11R was regulated by HOXC-AS3 and miR-216. Pearson’s correlation analysis for **(E)**; t-test for **(B, C, F, G)**; one-way ANOVA and *post hoc* test for **(H)**. **P < 0.01.

To explore the mechanism of miR-216, we predicted 24 possible downstream mRNAs using four bioinformatics tools: RNA22 (https://cm.jefferson.edu/rna22/Precomputed/), starBase, miRDB (http://mirdb.org/), and TargetScan (http://www.targetscan.org/vert_72/). (**Figure 3A**). We searched the expression level of candidate genes by GEPIA and found that only five genes (F11R, YBX1, BCAT1, IMPAD1, and RP2) were significantly upregulated in gliomas (**Figure S3** and **Table S5**). Subsequently, the changes in the mRNA expression levels after the miR-216 mimic/inhibitor transfection were examined by qRT–PCR, and we discovered that F11R expression was the most substantially downregulated/upregulated (**Figures 3B, C**). Additionally, we predicted the potential binding sites between miR-216 and F11R through a bioinformatic analysis using TargetScan (**Figure 3D**). The inverse correlation between miR-216 and F11R expression was also observed in Pearson correlation analysis (**Figure 3E**). The interaction between miR-216 and F11R was verified through a luciferase reporter assay, and results indicate that the overexpression of miR-216 significantly reduced the luciferase activity of the F11R-WT plasmid. However, no substantial change was detected in the F11R-MUT plasmid (**Figure 3F**). We collected clinical samples and detected the expression of F11R by qRT–PCR and immunohistochemistry. F11R is overexpressed in glioma and positively related to the grade of glioma, and similar results were obtained in the NHAs and glioma cell lines (**Figures 3G–I**). The western blot analysis further verified the above results (**Figure 3J**). These findings indicate that F11R is the target of miR-216.

**Figure 4:**
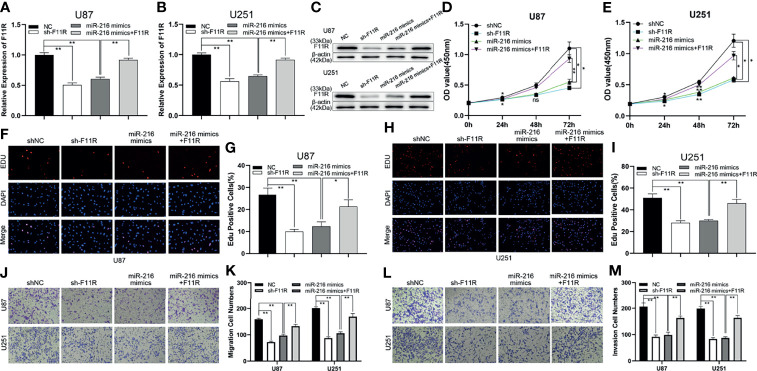
MiR-216 could inhibit glioma proliferation, migration, and invasion by targeting F11R. **(A–C)** qRT-PCR and western blot were used to test the expression of F11R in U87 and U251 cells transfected with NC, sh-F11R, miR-216 mimics, or miR-216 mimics together with F11R plasmids. **(D, E)** CCK-8 assay was used to test the proliferation of U87 and U251 cells transfected with NC, sh-F11R, miR-216 mimics, or miR-216 mimics together with F11R plasmids. **(F–I)** EdU assay was used to test the proliferation of U87 and U251 cells transfected with NC, sh-F11R, miR-216 mimics, or miR-216 mimics together with F11R plasmids. **(J, K)** Transwell assay was used to test the migration of U87 and U251 cells transfected with NC, sh-F11R, miR-216 mimics, or miR-216 mimics together with F11R plasmids. **(L, M)** Transwell assay was used to test the invasion of U87 and U251 cells transfected with NC, sh-F11R, miR-216 mimics, or miR-216 mimics together with F11R plasmids.One-way ANOVA and *post hoc* test for **(A, B, G, I, K, M)**. Two-way ANOVA and *post hoc* test for **(D, E)**. *P < 0.05, **P < 0.01.

To further explore the relationship between miR-216 and F11R, a shRNA against F11R (sh-F11R), miR-216 mimics, and miR-216 mimics along with the F11R plasmid were transfected into glioma cells. As shown by qRT–PCR and a western blot analysis, the expression of F11R was decreased by the sh-F11R and miR-216 mimics, and this inhibitory effect was partially recovered by the F11R plasmid (**Figures 4A–C**). We also obtained some interesting results. According to the EdU and CCK-8 assay results, the reduced proliferation caused by the miR-216 mimics was ameliorated by the transfection with the F11R plasmid (**Figures 4D–I**). The inhibitory effect of the miR-216 mimics on migration and invasion was reversed by the F11R plasmid (**Figures 4J–M**). Hence, we deduce that miR-216 inhibits glioma progression by regulating F11R expression.

### HOXC-AS3, as a ceRNA, Regulates F11R by Competitively Binding miR-216

**Figure 5:**
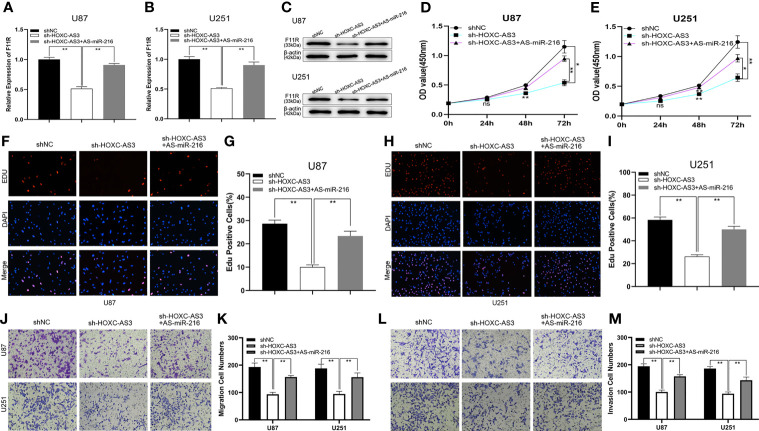
HOXC-AS3 promoted the glioma malignancy phenotype by targeting the miR-216/F11R axis. **(A–C)** qRT-PCR and western blot were used to test the expression of F11R in U87 and U251 cells transfected with NC, sh-HOXC-AS3, or sh-HOXC-AS3 together with miR-216 inhibitors. **(D, E)** CCK-8 assay was used to test the proliferation of U87 and U251 cells transfected with NC, sh-HOXC-AS3, or sh-HOXC-AS3 together with miR-216 inhibitors. **(F–I)** EdU assay was used to test the proliferation of U87 and U251 cells transfected with NC, sh-HOXC-AS3, or sh-HOXC-AS3 together with miR-216 inhibitors. **(J, K)** Transwell assay was used to test the migration of U87 and U251 cells transfected with NC, sh-HOXC-AS3, or sh-HOXC-AS3 together with miR-216 inhibitors. **(L, M)** Transwell assay was used to test the invasion of U87 and U251 cells transfected with NC, sh-HOXC-AS3, or sh-HOXC-AS3 together with miR-216 inhibitors. *P < 0.05, **P < 0.01. One-way ANOVA and *post hoc* test for **(A, B, G, I, K, M)**. Two-way ANOVA and *post hoc* test for **(D, E)**. *P < 0.05, **P < 0.01.

Current research suggests that HOXC-AS3 targets miR-216 as a sponge and that miR-216 regulates glioma progression by acting on F11R. To elucidate the HOXC-AS3-mediated ceRNA mechanism in glioma, the following rescue assays were conducted to analyze whether HOXC-AS3 modulates the expression of F11R in a miR-216-dependent manner. An shRNA against HOXC-AS3 (sh-HOXC-AS3), and sh-HOXC-AS3 along with miR-216 inhibitors were transfected into glioma cells. As shown by qRT–PCR and a western blot analysis, sh-HOXC-AS3 downregulated the expression of F11R, and the suppressive effect was partially counteracted by the miR-216 inhibitors (**Figures 5A–C**). Moreover, the effect of sh-HOXC-AS3 on glioma cell proliferation, migration, and invasion was also largely abrogated by the miR-216 inhibitor (**Figures 5D–M**). Overall, these results demonstrate that HOXC-AS3, as a ceRNA, regulates F11R by competitively binding miR-216.

### HOXC-AS3 Promotes Tumor Growth By Regulating miR-216 And F11R *In Vivo*

**Figure 6:**
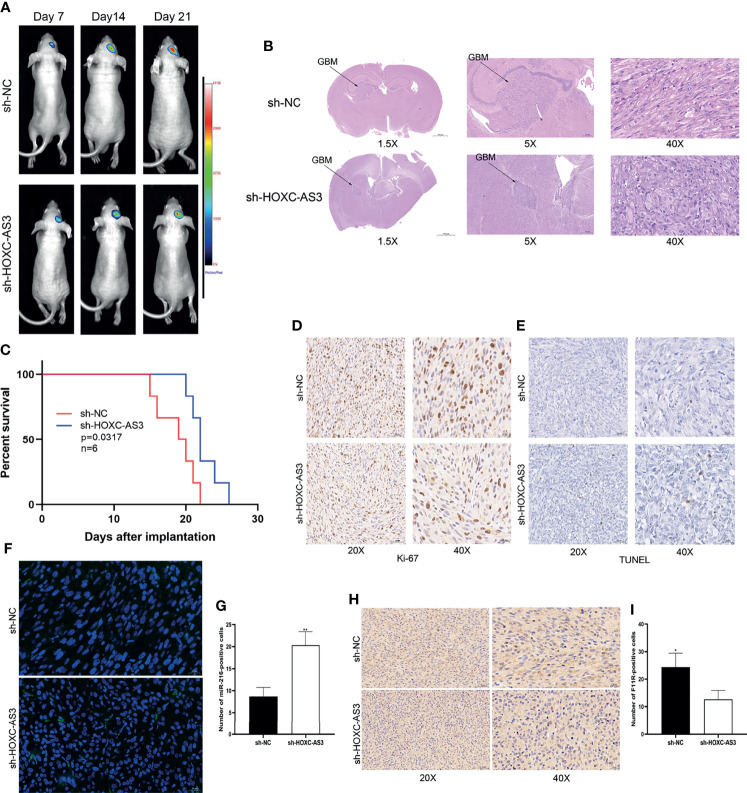
HOXC-AS3 promotes glioma growth *in vivo*. **(A)** Luciferase signals were assessed at 7,14 and 21 days after implantation of glioma cells (6 mice per group). **(B)** Tumor maximum diameter was determined using H&E staining. **(C)** Overall survival of the sh-HOXC-AS3 and control groups was compared by Kaplan-Meier survival curves. **(D)** Ki-67 expression in the sh-HOXC-AS3 and sh-NC groups was measured by immunohistochemistry. **(E)** TUNEL staining was used to detect cell apoptosis in the sh-HOXC-AS3 and sh-NC groups. **(F, G)** Expression and quantification of miR-216 in the brain sections. **(H, I)** Expression and quantification of F11R in the brain sections. Log-rank test for **(C)** and t-test for **(G, I)**. *P < 0.05, **P < 0.01.

To assess the oncogenic function of HOXC-AS3 *in vivo*, an orthotopic xenograft tumorigenicity assay was performed by stereotactic intracerebral injection of luciferase-expressing U87 cells (lacking HOXC-AS3 expression and negative control). The *in vivo* imaging conducted on the 7th, 14th, and 21st days after implantation showed that tumor growth in the sh-HOXC-AS3 group was significantly inhibited (**Figures 6A, B**). The survival rate of the mice injected with sh-HOXC-AS3-expressing cells was also elevated than that of the control mice (**Figure 6C**). The immunohistochemical results of the tumor sections showed that the expression level of Ki-67 in the sh-HOXC-AS3 group was substantially decreased; however, TUNEL staining was upregulated (**Figures 6D, E**). We further analyzed the expression of miR-216 and F11R in the brain sections, and the results indicated that silencing HOXC-AS3 raised the expression of miR-216 and suppressed F11R expression (**Figures 6F–I**). In conclusion, HOXC-AS3 facilitates glioma growth through regulating miR-216 and F11R expression *in vivo*.

## Discussion

Temozolomide (TMZ) is the most commonly used chemotherapy protocol for glioma treatment. Resistance to TMZ is the leading cause of inefficient chemotherapy (19). LncRNA CASC2 increased the sensitivity of glioma cells to TMZ by upregulating the expression of tumor suppressor PTEN through sponging miR-181a (20). LncRNA TALC was overexpressed in drug-resistant GBM cells, and TALC/miR-20b-3p/c-Met axis induced GBM chemotherapy resistance by promoting O6-methylguanine DNA methyltransferase (MGMT) function (21). Moreover, LncRNA SNHG12 was upregulated in TMZ-resistant tissues and cells. SNHG12 served as a sponge for miR-129-5p and raised E2F7 and MAPK1 expression. Silencing SNHG12 improved glioma cell sensitivity to TMZ (17). Therefore, LncRNA can directly affect sensitivity to TMZ, thus guiding the development of chemotherapeutic agents.

Exosomes are small vesicles secreted by cells, ranging in diameter from 40nm to 140nm, that play a vital role in intercellular signaling. Exosomes involved in various cellular and biological functions are the current research focus (22). Drug-resistant GBM cells could deliver SBF2-AS1 to drug-sensitive GBM cells through exosomes, leading to wide-spread chemotherapy resistance (23). Exosomes are small in size and can act as lncRNA carriers across the blood-brain barrier (BBB). Targeting exosome may be a promising treatment strategy (24). MiR-146b-overexpressing exosomes released by MSCs could significantly inhibit glioma growth in a primary glioma rat model (25). MSCs could also deliver anti-miR-9 exosomes to promote GBM sensitivity to TMZ (26). Marleau et al. proposed to use plasmapheresis technology combined with renal dialysis to selectively capture or retain exosomes in the circulatory system for subsequent treatment (27). However, the specificity of exosome packaging molecules and the mechanisms by which exosomes target cells or tissues need to be further researched, thus their clinical use is still challenging.

Recently, research has shown that lncRNAs play an indispensable role in the biological progression of various tumors. For instance, Li SY et al. reported that Lnc-APUE is restrained by HNF4α and facilitates hepatocellular carcinoma growth through the HNF4α/lnc-APUE/miR-20b/E2F1 axis (28). Chen C et al. showed that exosomal LNMAT2 facilitated lymphangiogenesis, lymphatic metastasis, HLEC tube formation, and migration by upregulating PROX1 expression in bladder cancer (29). Liu et al. demonstrated that lncRNA-HOTAIR sponges miR-126 as a ceRNA to facilitate glioma progression (30). In this research, we elucidated the mechanism of HOXC-AS3 in glioma progression.

By analyzing the differentially expressed lncRNA data in the TCGA database, we discovered that the expression of HOXC-AS3 in tumor tissues was significantly higher than that in normal tissues. HOXC-AS3 is a lncRNA that acts in many human tumors. For instance, Su J et al. reported that HOXC-AS3 can combine with YBX1 to directly transcriptionally activate TK1 and then regulate breast cancer progression (31). Fu T et al. found that HOXC-AS3 might regulate a series of HOX genes and has great value in the diagnosis of gastric adenocarcinoma (32). Li B et al. showed that HOXC-AS3 can interact with HOXC10, which increases the stability of HOXC10 and then promotes its expression, thereby reducing the osteogenic potential of Mesenchymal stem cells (MSCs) (33). These studies suggest that HOXC-AS3 may play a role as an oncogene in various tumors; however, the potential mechanism requires further investigation. In this study, we first confirmed the overexpression of HOXC-AS3 in both glioma tissues and cells. A high expression of HOXC-AS3 predicted a detrimental prognosis in glioma patients. The function of HOXC-AS3 on glioma cell proliferation, migration, invasion, and tumor growth *in vivo* was also examined.

Ample evidence has indicated that lncRNAs sponge miRNAs as ceRNAs and suppress the target binding of miRNAs to mRNAs, therefore modulating the expression of target mRNAs. Previous studies have shown that HOXC-AS3 can promote breast cancer metastasis by acting as a miR-3922-5p sponge (34). Our FISH assay identified that HOXC-AS3 is principally expressed in the cytoplasm of glioma cells. Based on these studies, HOXC-AS3 may function *via* a ceRNA mechanism in glioma. We subsequently probed the bound miRNA of HOXC-AS3 by a biological analysis, and seven miRNAs were predicted as targets of HOXC-AS3. MiR-216 was determined to be a target of HOXC-AS3 through qRT–PCR and a luciferase reporter analysis. It also has been shown to have an antitumor effect in various human tumors. For instance, Roscigno G et al. reported that miR-216a acts as a suppressor to reduce stem-like properties and influence the interaction between cells and the microenvironment in breast cancer (35). Qu XH et al. showed that miR-216 affects the growth, metastasis, and cell apoptosis of OSCC cells (36). Sun T et al. demonstrated that miR-216 inhibits the cell progression of ESCC through the miR-216/KIAA0101 axis (37). Wang W et al. found that miR-216a suppressed glioma cell progression and promoted apoptosis through the miR-216a/LGR5 axis (38). In this research, we demonstrate that miR-216 could interact with HOXC-AS3 and that the expression of miR-216 was negatively correlated with HOXC-AS3 expression in glioma cells and clinical samples.

Subsequently, the target mRNA of miR-216 was examined. F11R is a functional target of miR-216. In previous studies, F11R was originally identified as a monoclonal antibody receptor (39) that acts as a tight junction protein and is expressed in several leukocyte populations and platelets (40, 41). A series of studies revealed that the expression of F11R is inversely correlated with prognosis in many tumors, such as multiple myeloma (42), oral squamous cell carcinoma (43), and epithelial ovarian cancer (44). F11R is overexpressed in tumor tissues and promotes biological processes in HNSCC (45). However, in some tumors, a low expression of F11R may lead to a poor prognosis; for example, Communal L et al. showed that expression of F11R is a reliable prognostic biomarker of HGSC that may be used to distinguish tumors that respond to EMT therapy. The decreased F11R gene indicates a poor outcome (46). F11R can also act as a tumor suppressor gene in anaplastic thyroid carcinoma (47) and nasopharyngeal carcinoma (48). In glioma cancer stem cells (CSCs), F11R functions to promote tumor initiation, cell proliferation, and self-renewal *in vitro*, and F11R is suppressed by miR-145 (49). Rosager AM et al. reported that F11R is colocalized with stem cell markers and that the expression of F11R is positively correlated with the grade of glioma. In GBMs, F11R is an independent prognostic factor (50, 51). In the female tumor microenvironment, F11R inhibits pathogenic microglial activation and indicates sex differences in glioma initiation (52). However, its biological function has not been described in detail. In this study, we discovered that the expression of F11R increased with the grade of glioma and that F11R promoted malignant progression downstream of miR-216. We also performed rescue experiments, and the results showed that the effect of miR-216 mimics was abolished by F11R plasmids and that the function of sh-HOXC-AS3 could be reversed by miR-216 inhibitors in glioma cells.

In conclusion, HOXC-AS3 facilitates glioma progression *via* miR-216 to regulate F11R. Hence, the HOXC-AS3/miR-216/F11R signaling pathway may provide a potential target for the treatment of glioma. HOXC-AS3 may become a target to improve TMZ sensitivity, or regulate glioma tumorigenesis through exosome packaging, which requires further research.

## Data Availability Statement

The datasets presented in this study can be found in online repositories. The names of the repository/repositories and accession number(s) can be found in the article/**Supplementary Material**.

## Ethics Statement

The studies involving human participants were reviewed and approved by the institute ethical committee of the Affiliated Brain Hospital of Nanjing Medical University. The patients/participants provided their written informed consent to participate in this study. The animal study was reviewed and approved by the Institutional Animal Care and Use Committee of Nanjing Medical University.

## Author Contributions

CQ and HL designed and funded the study. YL, LP, and XC performed experiments and wrote the paper. KY collected clinical specimens. ZW directed and participated in animal experiments. HX contributed suitable reagents or analytic tools. YX conducted bioinformatics analysis to guide the experiment. All authors read and approved the final manuscript.

## Funding

National Natural Science Foundation of China (81972350). This research was funded by the National Natural Science Foundation of China (81972350).

## Conflict of Interest

The authors declare that the research was conducted in the absence of any commercial or financial relationships that could be construed as a potential conflict of interest.

## Publisher’s Note

All claims expressed in this article are solely those of the authors and do not necessarily represent those of their affiliated organizations, or those of the publisher, the editors and the reviewers. Any product that may be evaluated in this article, or claim that may be made by its manufacturer, is not guaranteed or endorsed by the publisher.

## Supplementary Material

Supplementary Figure 1 Downregulation of HOXC-AS3 inhibits proliferation, migration, and invasion of glioma *in vitro*. **(A, B)** qRT-PCR was used to test the expression of HOXC-AS3 in U87 and U251 cells transfected with shNC, sh-HOXC-AS3-1, or sh-HOXC-AS3-2. **(C, D)** CCK-8 assay was used to test the proliferation of U87 and U251 cells transfected with shNC, sh-HOXC-AS3-1, or sh-HOXC-AS3-2. **(E**–**H)** EdU assay was used to test the proliferation of U87 and U251 cells transfected with shNC, sh-HOXC-AS3-1, or sh-HOXC-AS3-2. **(I, J)** Transwell assay was used to test the migration of U87 and U251 cells transfected with shNC, sh-HOXC-AS3-1, or sh-HOXC-AS3-2. **(K, L)** Transwell assay was used to test the invasion of U87 and U251 cells transfected with shNC, sh-HOXC-AS3-1, or sh-HOXC-AS3-2. One-way ANOVA and *post hoc* test for **(A, B, F, H, J, L)**. Two-way ANOVA and *post hoc* test for **(C, D)**. *P < 0.05, **P < 0.01.

Supplementary Figure 2 Upregulation of HOXC-AS3 promotes proliferation, migration, and invasion of glioma *in vitro*. **(A, B)** qRT-PCR was used to detect the expression of HOXC-AS3 in U87 and U251 cells transfected with NC or HOXC-AS3 plasmid. **(C, D)** CCK-8 assay was used to detect the proliferation of U87 and U251 cells transfected with NC or HOXC-AS3 plasmid. **(E**–**H)** EdU assay was used to detect the proliferation of U87 and U251 cells transfected with NC or HOXC-AS3 plasmid. **(I, J)** Transwell assay was used to detect the migration of U87 and U251 cells transfected with NC or HOXC-AS3 plasmid. **(K, L)** Transwell assay was used to detect the invasion of U87 and U251 cells transfected with NC or HOXC-AS3 plasmid. T-test for **(A, B, F, H, J, L)**. Two-way ANOVA and *post hoc* test for **(C, D)**. *P < 0.05, **P < 0.01.

Supplementary Figure 3 The expression of 24 genes downstream of miR-216 was predicted by GEPIA.

Supplementary Figure 4 Quantification and statistical analysis of the tumors in xenograft mouse model. T-test was used. *P < 0.05, **P < 0.01.

Supplementary Figure 5 Median survival analysis of xenograft mouse model. Log-rank test was used. *P < 0.05, **P < 0.01.

Supplementary Figure 6 Statistical analysis of the three random fields of the expression level of Ki-67 and TUNEL staining. T-test was used. *P < 0.05, **P < 0.01.
