# Supplementary figures and images for: The Long Non-Coding RNA HOXC-AS3 Promotes Glioma Progression by Sponging miR-216 to Regulate F11R Expression
Source: Front Oncol. 2022 Mar 23;12:845009. doi: 10.3389/fonc.2022.845009 (PMC8984117; doi:10.3389/fonc.2022.845009)

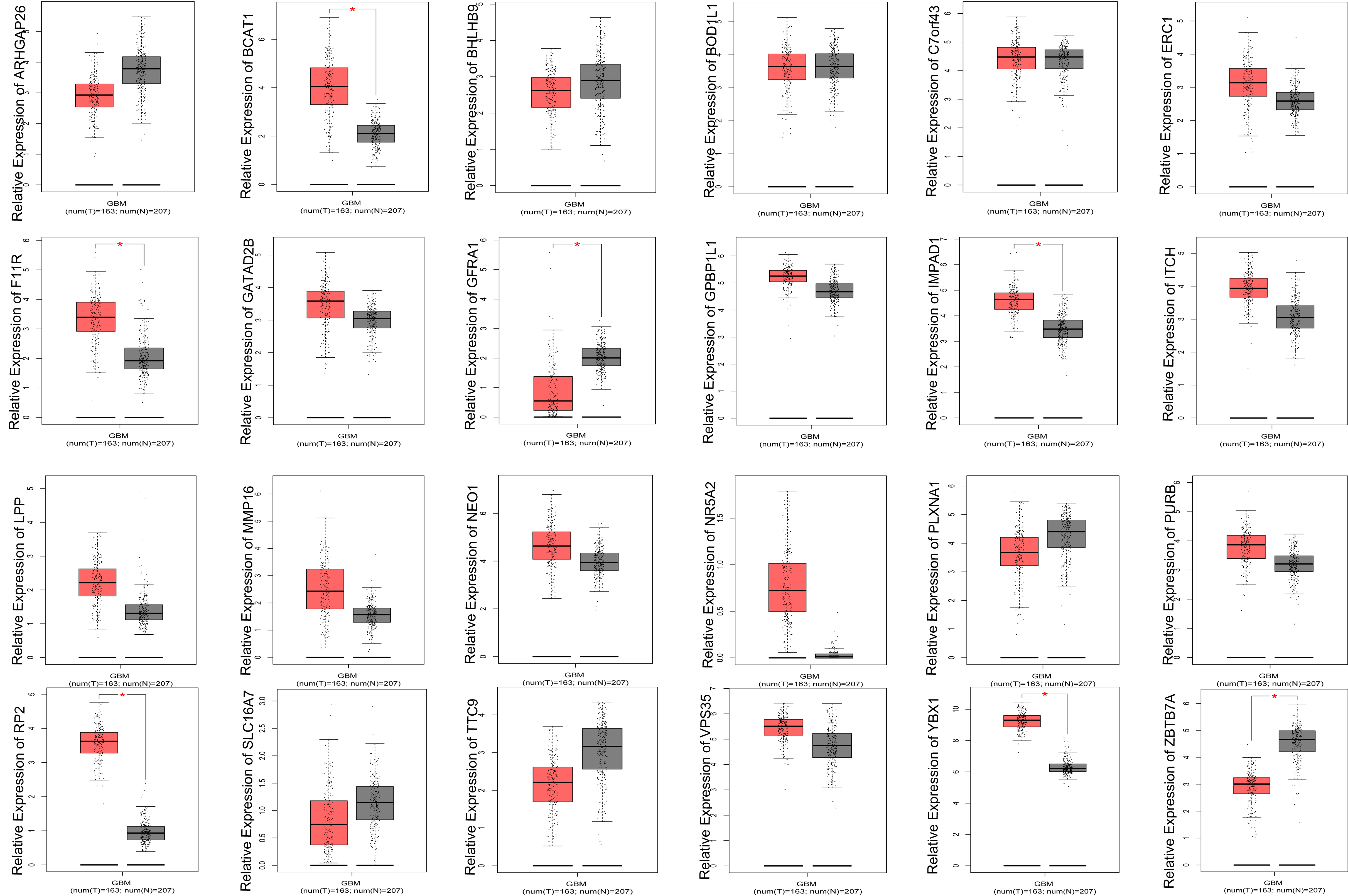

Supplement: Supplementary Figure 1 — Downregulation of HOXC-AS3 inhibits proliferation, migration, and invasion of glioma in vitro. (A, B) qRT-PCR was used to test the expression of HOXC-AS3 in U87 and U251 cells transfected with shNC, sh-HOXC-AS3-1, or sh-HOXC-AS3-2. (C, D) CCK-8 assay was used to test the proliferation of U87 and U251 cells transfected with shNC, sh-HOXC-AS3-1, or sh-HOXC-AS3-2. (E–H) EdU assay was used to test the proliferation of U87 and U251 cells transfected with shNC, sh-HOXC-AS3-1, or sh-HOXC-AS3-2. (I, J) Transwell assay was used to test the migration of U87 and U251 cells transfected with shNC, sh-HOXC-AS3-1, or sh-HOXC-AS3-2. (K, L) Transwell assay was used to test the invasion of U87 and U251 cells transfected with shNC, sh-HOXC-AS3-1, or sh-HOXC-AS3-2. One-way ANOVA and post hoc test for (A, B, F, H, J, L). Two-way ANOVA and post hoc test for (C, D). *P < 0.05, **P < 0.01. [file Image_1.tif]

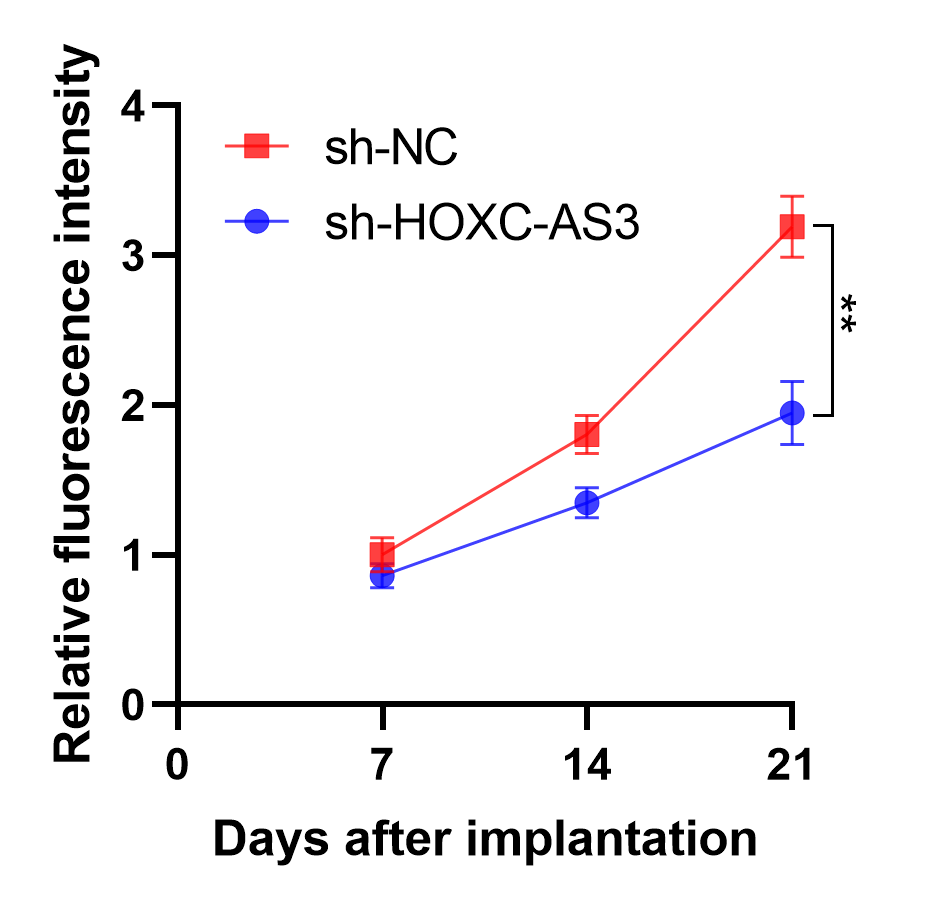

Supplement: Supplementary Figure 2 — Upregulation of HOXC-AS3 promotes proliferation, migration, and invasion of glioma in vitro. (A, B) qRT-PCR was used to detect the expression of HOXC-AS3 in U87 and U251 cells transfected with NC or HOXC-AS3 plasmid. (C, D) CCK-8 assay was used to detect the proliferation of U87 and U251 cells transfected with NC or HOXC-AS3 plasmid. (E–H) EdU assay was used to detect the proliferation of U87 and U251 cells transfected with NC or HOXC-AS3 plasmid. (I, J) Transwell assay was used to detect the migration of U87 and U251 cells transfected with NC or HOXC-AS3 plasmid. (K, L) Transwell assay was used to detect the invasion of U87 and U251 cells transfected with NC or HOXC-AS3 plasmid. T-test for (A, B, F, H, J, L). Two-way ANOVA and post hoc test for (C, D). *P < 0.05, **P < 0.01. [file Image_2.tif]

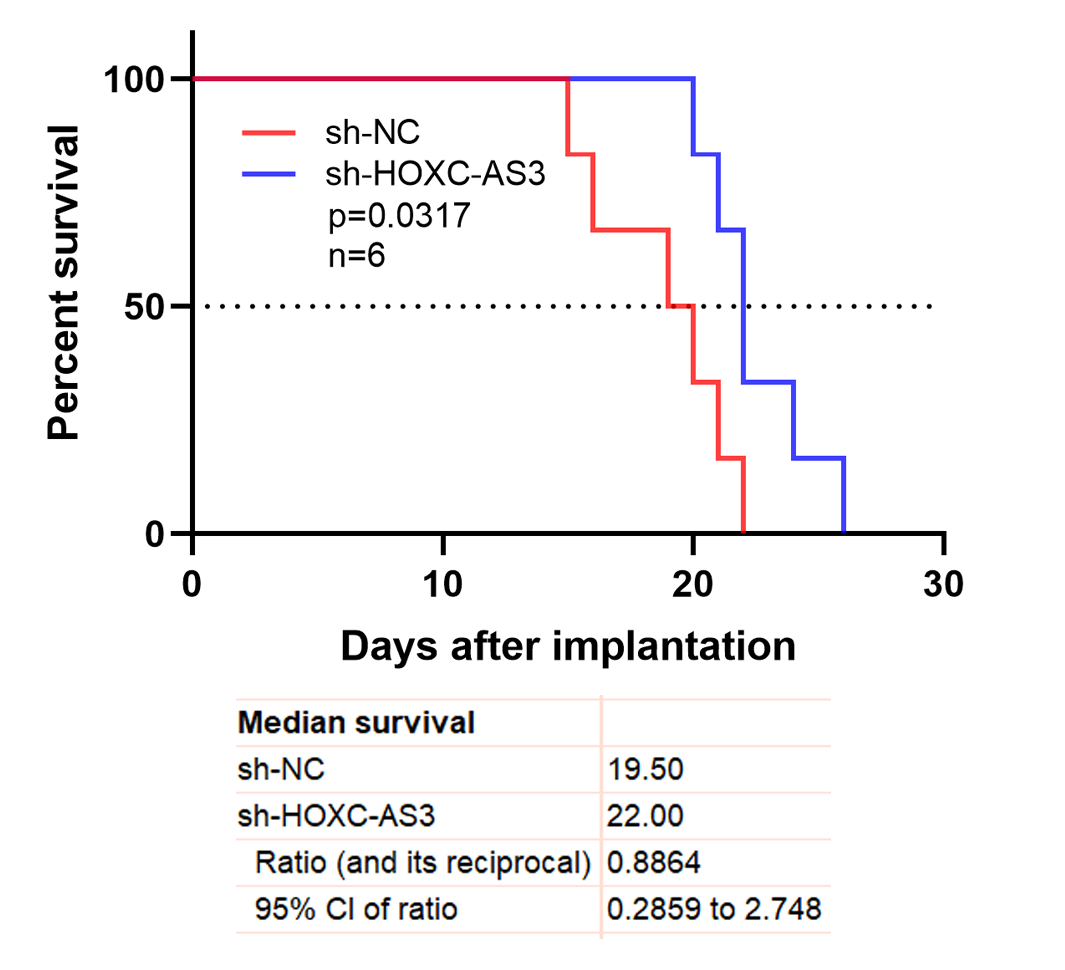

Supplement: Supplementary Figure 3 — The expression of 24 genes downstream of miR-216 was predicted by GEPIA. [file Image_3.tif]

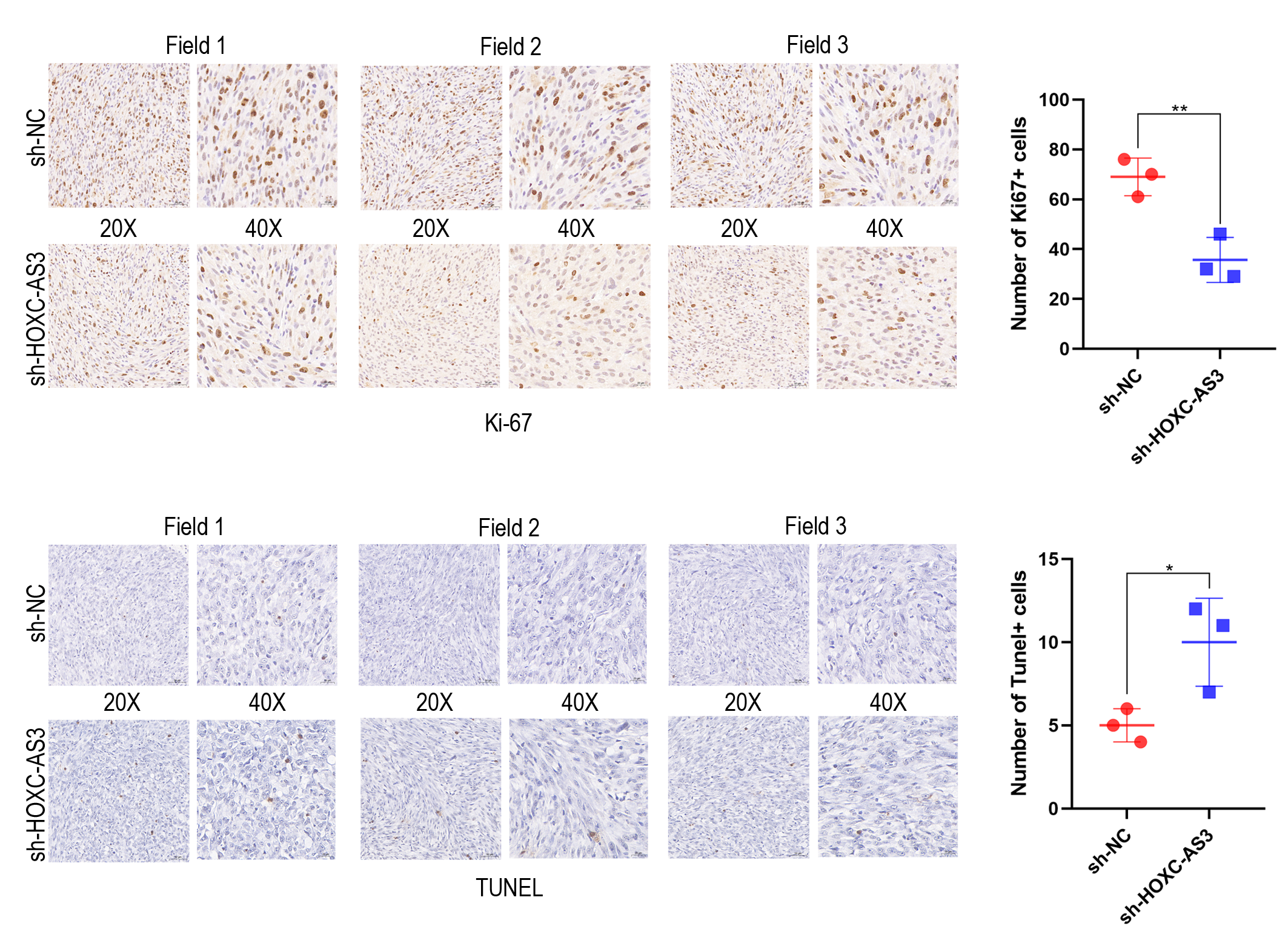

Supplement: Supplementary Figure 4 — Quantification and statistical analysis of the tumors in xenograft mouse model. T-test was used. *P < 0.05, **P < 0.01. [file Image_4.tif]

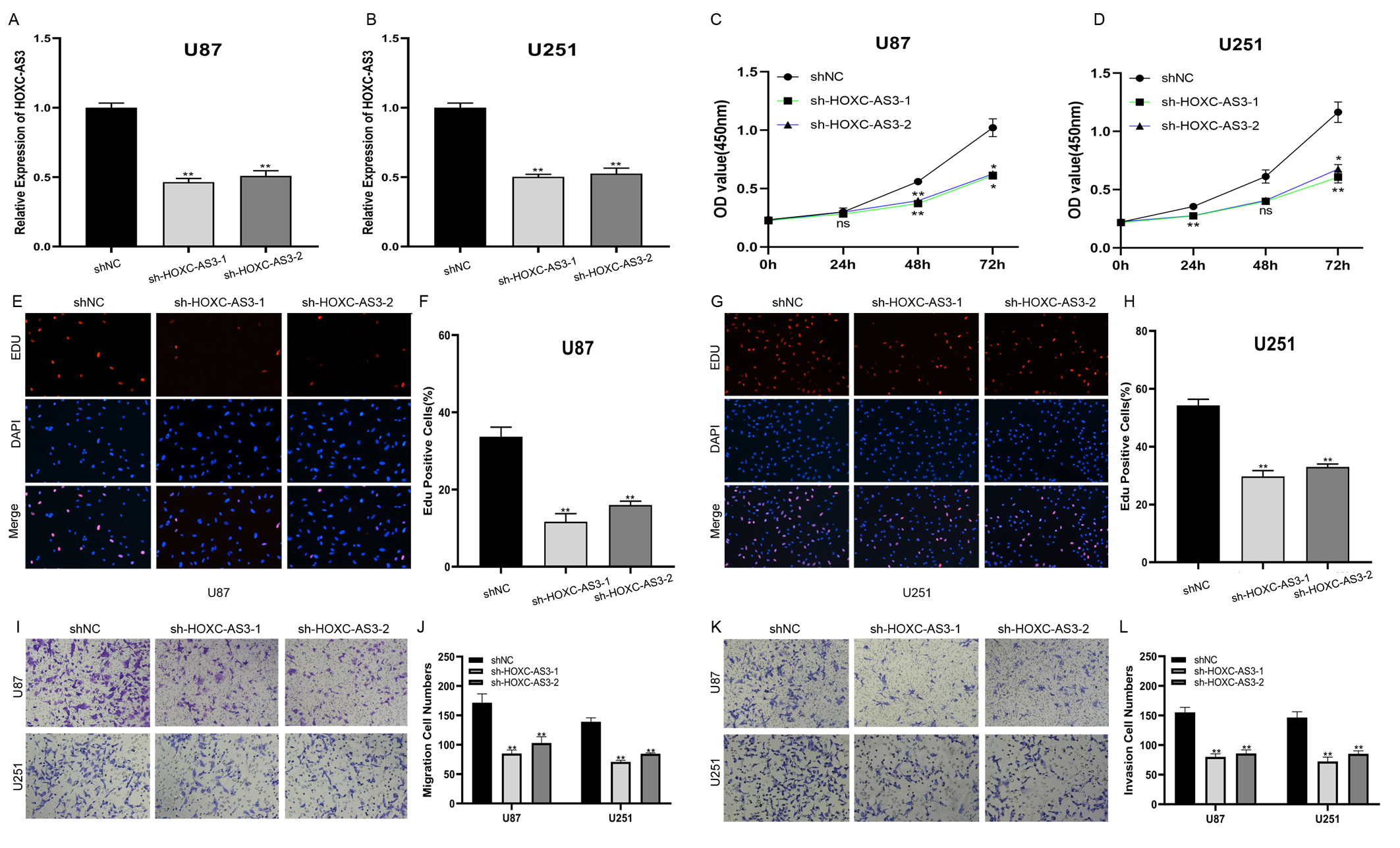

Supplement: Supplementary Figure 5 — Median survival analysis of xenograft mouse model. Log-rank test was used. *P < 0.05, **P < 0.01. [file Image_5.tif]

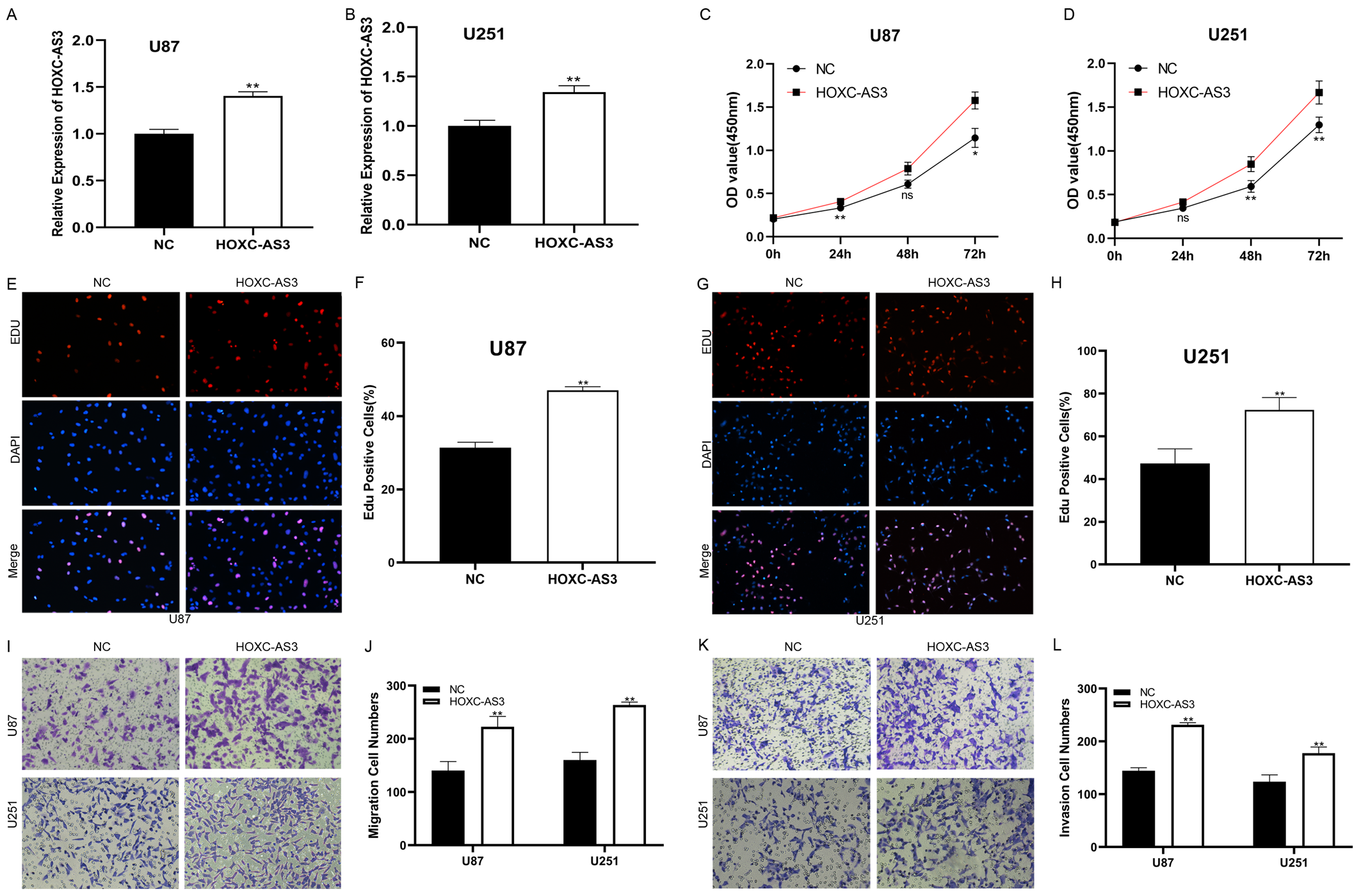

Supplement: Supplementary Figure 6 — Statistical analysis of the three random fields of the expression level of Ki-67 and TUNEL staining. T-test was used. *P < 0.05, **P < 0.01. [file Image_6.tif]
