# Supplementary material for: The Long Non-Coding RNA HOXC-AS3 Promotes Glioma Progression by Sponging miR-216 to Regulate F11R Expression
Source: Front Oncol. 2022 Mar 23;12:845009. doi: 10.3389/fonc.2022.845009 (PMC8984117; doi:10.3389/fonc.2022.845009)
Supplement: Supplementary file 7 [file Table_1.docx]

**Table S1:** **The interfering nucleotide used in this study**

| **Name** | **Sequences (5’-3’)** |
| --- | --- |
| sh-HOXC-AS3-1 | CCCAGAGAAGCGTCCTTTA |
| sh-HOXC-AS3-2 | TCACGTATCACACGGGAAATT |
| hsa-miR-216 mimics | Sense:UAAUCUCAGCUGGCAACUGUGA |
|  | Antisense:ACAGUUGCCAGCUGAGAUUAUU |
| mimics N.C | Sense:UUGUACUACACAAAAGUACUG |
|  | Antisense:GUACUUUUGUGUAGUACAAUU |
| hsa-miR-216 inhibitor | UCACAGUUGCCAGCUGAGAUUA |
| Inhibitor NC | CAG UAC UUU UGU GUA GUA CAA |
| sh-F11R | GGAAACTGTTGTGCCTCTTCA |
| Sh-NC | TTCTCCGAACGTGTCACGT |
