# Supplementary material for: The Long Non-Coding RNA HOXC-AS3 Promotes Glioma Progression by Sponging miR-216 to Regulate F11R Expression
Source: Front Oncol. 2022 Mar 23;12:845009. doi: 10.3389/fonc.2022.845009 (PMC8984117; doi:10.3389/fonc.2022.845009)
Supplement: Supplementary file 8 [file Table_2.docx]

**Table S2: The primers used in this study**

| **Gene name** | **Sequences** |
| --- | --- |
| HOXC-AS3 | F：5′-GTGGAGTAACAGCGCCATCT-3′ |
|  | R：5′-CGGGTTTTGTTGCGTCTTGT-3′ |
| hsa-miR-497 | F：5′-CGGGCCAGCAGCACACTGT-3′ |
|  | R：5′-CAGCCACAAAAGAGCACAAT-3′ |
| hsa-miR-15a | F：5′-CGGGCTAGCAGCACATAATG-3′ |
|  | R：5′-CAGCCACAAAAGAGCACAAT-3′ |
| hsa-miR-15b | F：5′-CGGGCTAGCAGCACATCATG-3′ |
|  | R：5′-CAGCCACAAAAGAGCACAAT-3′ |
| hsa-miR-424 | F：5′-CGGGCCAGCAGCAATTCATG-3′ |
|  | R：5′-CAGCCACAAAAGAGCACAAT-3′ |
| hsa-miR-195 | F：5′-CGGGCTAGCAGCACAGAAA-3′ |
|  | R：5′-CAGCCACAAAAGAGCACAAT-3′ |
| hsa-miR-16 | F：5′-CGGGCTAGCAGCACGTAAAT-3′ |
|  | R：5′-CAGCCACAAAAGAGCACAAT-3′ |
| hsa-miR-216 | F：5′-CGGGCTAATCTCAGCTGGCA-3′ |
|  | R：5′-CAGCCACAAAAGAGCACAAT-3′ |
| F11R | F：5′-AAGGAGACACCACCAGACT-3′ |
|  | R：5′-AGGCATCACTATCCCATC-3′ |
| YBX1 | F：5′-GAGAAGTGATGGAGGGTGCT-3′ ; |
|  | R：5′-TTAGGGTTTTCTGGGCGTCT-3′ |
| BCAT1 | F：5′-TGTCTCAAGGTTTCTGGTCA-3′; |
|  | R：5′-GGTGTGGTTTCTGGTCTTTC-3′ |
| GADPH | F：5′-GAAATCCCATCACCATCTTCCAGG-3′ ； |
|  | R：5′-GAGCCCCAGCCTTCTCCATG-3′ |
| U6 | F：5′-CTCGCTTCGGCAGCACA-3′ |
|  | R：5′-AACGCTTCACGAATTTGCGT-3′ |
