# Supplementary material for: The Long Non-Coding RNA HOXC-AS3 Promotes Glioma Progression by Sponging miR-216 to Regulate F11R Expression
Source: Front Oncol. 2022 Mar 23;12:845009. doi: 10.3389/fonc.2022.845009 (PMC8984117; doi:10.3389/fonc.2022.845009)
Supplement: Supplementary file 9 [file Table_3.docx]

**Table S3 Luciferase reporter assay sequences**

| **Name** | **Sequences(5'-3')** |
| --- | --- |
| HOXC-AS3-hsa-miR-216 wt | GGACGCAGACCTGAATAACTTGTTGGGTTTTCACAGACTTTGAGATTCTGCTTCAGTGGAGGTAAAAGGAAGGAA |
| HOXC-AS3-hsa-miR-216 MUT | GGACGCAGACCTGAATAACTTGTTGGGTTAACAGTCACTAACTCTAACTGCTTCAGTGGAGGTAAAAGGAAGGAA |
| F11R-hsa-miR-216 wt | TTCTTAGCTCTTTGGTGAGATTG |
| F11R-hsa-miR-216 MUT | TTCTTAGCTCTTTGGGTCTCGGG |
