# Supplementary material for: The Long Non-Coding RNA HOXC-AS3 Promotes Glioma Progression by Sponging miR-216 to Regulate F11R Expression
Source: Front Oncol. 2022 Mar 23;12:845009. doi: 10.3389/fonc.2022.845009 (PMC8984117; doi:10.3389/fonc.2022.845009)
Supplement: Supplementary file 10 [file Table_4.docx]

**Table. S4 FISH probe**

| Name |  | Sequences(5'-3') |
| --- | --- | --- |
|  |  |  |
| HOXC-AS3 homo probe mixs | HOXC-AS3 homo probe | CTCCGCAGGT+TCCCTCT+TTCTTTCC |
|  | HOXC-AS3 homo probe1 | TCT+TCT+TTCAAACATGCTCCCAGCCC |
|  | HOXC-AS3 homo probe2 | CTAGA+TGGCGCTGT+TACTCCACTCTGC |
| miR-216 probe |  | TCACTGTTGCCTGCTGAGATTA |
