# Supplementary material for: The Long Non-Coding RNA HOXC-AS3 Promotes Glioma Progression by Sponging miR-216 to Regulate F11R Expression
Source: Front Oncol. 2022 Mar 23;12:845009. doi: 10.3389/fonc.2022.845009 (PMC8984117; doi:10.3389/fonc.2022.845009)
Supplement: Supplementary file 11 [file Table_5.docx]

**Table. S5 The list of 24 mRNAs which may be biological targets of miR-216**

| **Candidate mRNAs** | **Full name** |
| --- | --- |
| GPBP1L1 | GC-rich promoter binding protein 1 like 1 |
| GATAD2B | GATA zinc finger domain containing 2B |
| F11R | F11 receptor |
| YBX1 | Y-box binding protein 1 |
| NR5A2 | nuclear receptor subfamily 5 group A member 2 |
| GFRA1 | GDNF family receptor alpha 1 |
| BCAT1 | branched chain amino acid transaminase 1 |
| ERC1 | ELKS/RAB6-interacting/CAST family member 1 |
| SLC16A7 | solute carrier family 16 member 7 |
| TTC9 | tetratricopeptide repeat domain 9 |
| NEO1 | neogenin 1 |
| VPS35 | VPS35 retromer complex component |
| ZBTB7A | zinc finger and BTB domain containing 7A |
| ITCH | itchy E3 ubiquitin protein ligase |
| PLXNA1 | plexin A1 |
| LPP | LIM domain containing preferred translocation partner in lipoma |
| BOD1L1 | biorientation of chromosomes in cell division 1 like 1 |
| ARHGAP26 | Rho GTPase activating protein 26 |
| PURB | purine rich element binding protein B |
| C7orf43 | trafficking protein particle complex subunit 14 |
| IMPAD1 | 3'(2'), 5'-bisphosphate nucleotidase 2 |
| MMP16 | matrix metallopeptidase 16 |
| RP2 | RP2 activator of ARL3 GTPase |
| BHLHB9 | basic helix-loop-helix family member b9 |
