# Supplementary material for: The Long Non-Coding RNA HOXC-AS3 Promotes Glioma Progression by Sponging miR-216 to Regulate F11R Expression
Source: Front Oncol. 2022 Mar 23;12:845009. doi: 10.3389/fonc.2022.845009 (PMC8984117; doi:10.3389/fonc.2022.845009)
Supplement: Supplementary file 12 [file DataSheet_1.pdf]

Cell Line Authentication Report

GENEWIZ,Inc. Beijing
30 Science Park Road
Zhong-Guan-Cun Life Science Park
Changping District, 102206
Beijing, China
Tel: 400-8100-669
Fax: 010-59458058
Email: Genomics.China@genewiz.com.cn

www.genewiz.com.cn

GENEWIZ,Inc. Beijing
30 Life Science Park Road
Changping District, 102206
Beijing, China
Tel: 400-8100-669
Fax: 010-59458058
www.genewiz.com.cn

Cell Line Authentication Report

Customer: HuYaLing

Institution: WUXI PEOPLE’S HOSPITAL

Quotation Number: M10377

Completion Date：03/14/2017

1. **Sample ID: KJ-U87**
2. **Original Material: Cell pellets**
3. **Methods:**

1).Genomic DNA was extracted from the cell pellets provided by the customer.

2).Samples, together with positive and negative control were amplified using GenePrint 10 System(Promega).

3). Amplified products were processed using the ABI3730xl Genetic Analyzer.

4).Datawere analyzed using GeneMapper4.0 software and then compared with the ATCC, DSMZ or JCRB databases for reference matching.

4. **Results:**

1) 10 Loci STR Profile：

Genetic Site	Customer sample	
(Locus)	KJ-U87	
Amelogenin	X	
CSF1PO	10	11
D13S317	8	11
D16S539	12	
D5S818	11	12
D7S820	8	9
THO1	9.3	
TPOX	8	
vWA	15	17
D21S11	28	32.2

<<<If the Percent match is not 100%, search for reference matchingwith the ATCC, DSMZ or JCRB databases and add the match results.

Addendum: Comparative output from the ATCC STR Profile database

Result of STR matching analysis by your data.

- DSMZ Profile Database -

A graphical presentation is shown at the bottom of this page.

EV	Cell No.	Cell name	Locus names									Figures
			D5S818	D13S317	D7S820	D16S539	VWA	TH01	AM	TPOX	CSF1PO	
	Query (Your Cell)		*11,12*	*8,11*	*8,9*	*12,12*	*15,17*	*9.3,9.3*	*X,X*	*8,8*	*10,11*	
1.00(36/36)	HTB-14	U-87MG	11,12	8,11	8,9	12,12	15,17	9.3,9.3	X,X	8,8	10,11	-
0.72(26/36)	731	CAKI-1	11,12	11,11	8,12	12,12	15,17	6,8	X,X	8,11	10,11	-
0.72(26/36)	749	U-CH2	10,11	11,11	8,12	12,12	17,17	9.3,9.3	X,X	8,8	11,12	-
0.72(26/36)	CRL-5842	NCI-H774 [H774]	11,11	8,8	9,11	12,12	15,17	6,9.3	X,X	8,8	10,10	-
0.72(26/36)	CRL-5910	NCI-H1994 [H1994]	10,11	11,11	9,11	12,12	15,19	7,9.3	X,X	8,8	10,11	-
0.72(26/36)	CRL-7064	Hs 94.T	11,12	12,13	8,9	9,12	17,18	7,9.3	X,X	8,8	10,11	-
0.72(26/36)	CRL-7935	"R1192 [BE; BEAN; R11 "	11,12	11,12	8,12	12,12	15,17	6,8	X,X	8,11	10,11	-
0.72(26/36)	HTB-46	Caki-1	11,12	11,12	8,12	12,12	15,17	6,8	X,X	8,11	10,11	-
0.72(26/36)	JCRB0801	Caki-1	11,12	11,12	8,12	12,12	15,17	6,8	X,X	8,11	10,11	-
0.72(26/36)	RCB1985	Caki-1	11,12	11,12	8,12	12,12	15,17	6,8	X,X	8,11	10,11	-

>>>

GENEWIZ,Inc. Beijing
30 Life Science Park Road
Changping District, 102206
Beijing, China
Tel: 400-8100-669
Fax: 010-59458058
www.genewiz.com.cn

2) Electrophoretogram

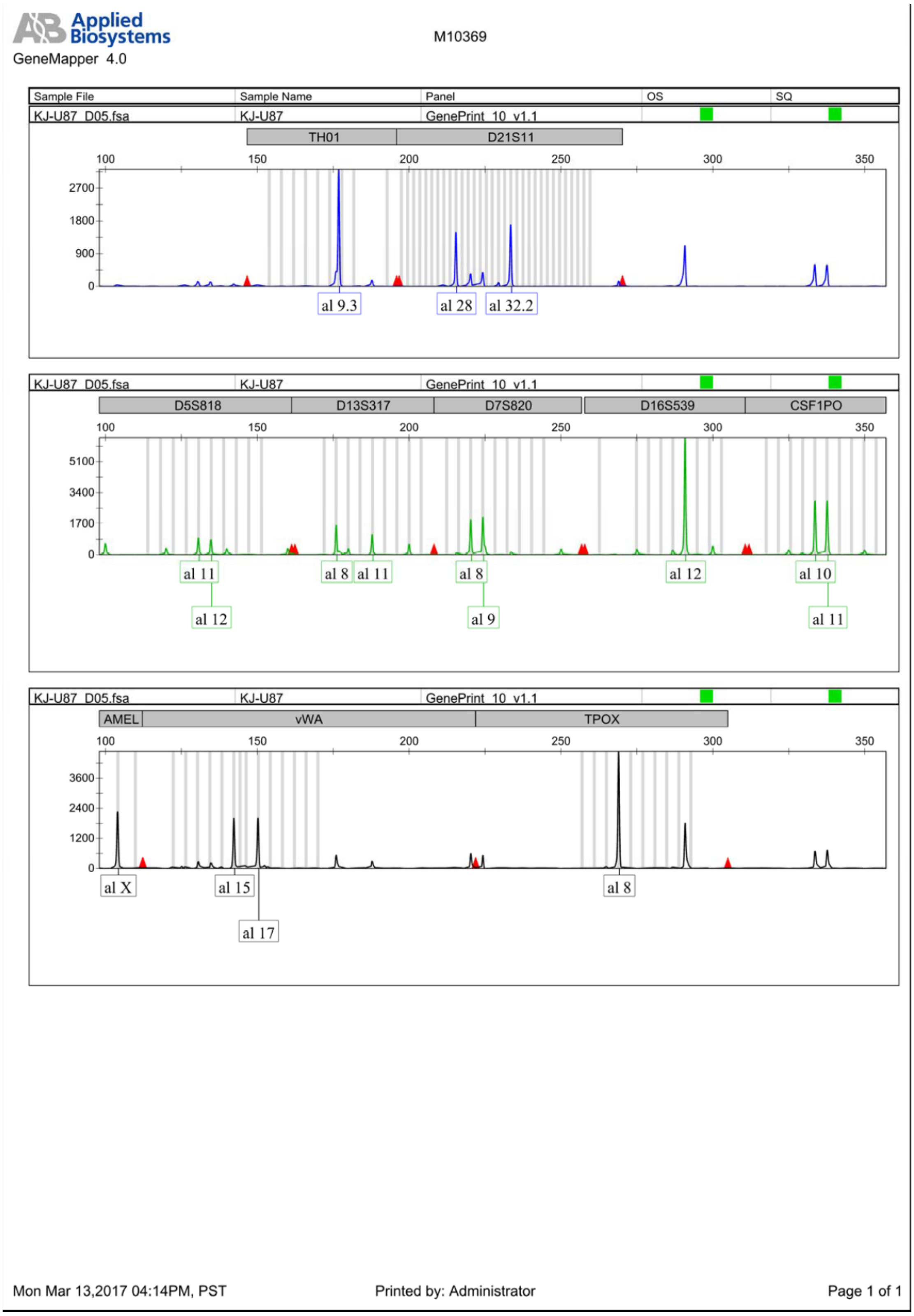

Note：Raw data in appendix
